# Malpractice Awareness among Surgeons and Surgical Trainees in Ethiopia

**DOI:** 10.4314/ejhs.v32i1.13

**Published:** 2022-01

**Authors:** Yonas Ademe, Andualem Deneke, Abebe Bekele

**Affiliations:** 1 Addis Ababa University, College of Health Sciences, School of Medicine, Department of Surgery, Addis Ababa, Ethiopia; 2 University of Global Health Equity, School of Medicine, Kigali Heights, Plot 772, KG 7 AVE

**Keywords:** malpractice, negligence, surgeon, knowledge, attitudes, practices

## Abstract

**Background:**

In Ethiopia, a country where seeing medical errors is not rare, there is a lack of data concerning the overall awareness of medical malpractice issues among physicians. A recent study showed that 80% of malpractice claims in Ethiopia are related to some form of surgery or operation room activities.

**Methods:**

A cross-sectional survey was conducted among surgeons and surgical trainees. Data were collected anonymously by an online survey using Google forms through a 56-items structured questionnaire. Subsequently, the data were analyzed and reported employing nonparametric statistical methods with SPSS software package 26.

**Results:**

In our sample, the overall awareness regarding medical malpractice was relatively low. Surgery on a wrong patient (71.1%) was the most commonly reported form of malpractice, whereas unintended damage to adjacent organs (10.8%) was the most frequently disagreed up on form. In the event of a medical error, the majority (59.6%) reported readiness to disclose their error to the patient. The most common mentioned reason for not revealing a mistake was a threat of physical or verbal assault (68%). A significant number of respondents, i.e., 120(59.1%), reported being physically/verbally assaulted by a patient or their attendants at some point in their practice.

**Conclusion:**

The findings of our study provided a general picture of surgeons' and surgical trainees' knowledge, attitude, and practice regarding medical malpractice. This study recommends more robust ethics and law training modules to surgical trainees, refresher courses to surgeons, and advanced training programs in ethics and law.

## Introduction

To Begin with the definition “Medical malpractice is professional negligence by a healthcare provider in which the treatment provided falls below the accepted standard of practice in the medical community and causes injury or death to the patient, with most cases involving medical error” (1). And hence, surgical malpractice is a form of medical malpractice occurring in surgical patients. It is believed that the majority of surgical adverse events are due to technical errors, but much is not known about the prevalence, types and reasons of these events (2).

Nearly 1 in every 10 patients admitted to a hospital will go through an adverse event (7). The overall prevalence of adverse medical events is reported to be comparable in developed and developing countries, ranging between 3.7% to 16.6% in developed countries and between 2.5% to 18.4% in developing countries (3–5). Malpractice claims were particularly found to be higher in surgical specialties with as many as one half to two thirds of inpatient adverse events resulting from inappropriate surgical care (6,7,14).

Studies have showed that medical errors account for a significant proportion of in-hospital mortality in both developed and developing countries. In USA, the report of the Institute of Medicine showed that around 44,000 to 98,000 hospitalized patients die each year due to preventable medical error; the 5th most common cause of death in USA, and this was more than deaths due to motor vehicle accidents, breast cancer, and AIDS (8). Although there is limited data on deaths due to medical errors in developing countries, a study reported that mortality due to medical errors is quite high (9).

In Ethiopia, there hasn't been a study done to assess the overall prevalence and morbidity and mortality of medical errors. However, a study analyzing medical malpractice claims to the Federal Health Professionals Ethics Committee showed that 80% of malpractice claims are related to some form of surgery or operation room activities. The majority of complaints were found to be against obstetricians/gynecologists (56.8%), followed by general surgeons (20.45%) and orthopedic surgeons (11.36%). The analysis also revealed that there is a rising trend in malpractice claims year after year (9).

Studies have shown a generally dismal level of awareness of physicians to medical malpractice and ethical concepts (19,29). However, there appears to be paucity of local data on the awareness of medical malpractice among surgeons or surgical trainees. This study is a cross-sectional survey conducted to investigate surgical practitioners' knowledge, attitude, and experience regarding surgical malpractice. The objective of the study is to look in to the overall awareness of surgeons and trainees on the current situation of medical malpractice in Ethiopia. It also tries to understand their perceptions and their awareness on legal provisions and their experiences with medical malpractice issues. This study is unique in the sense that no reports have been previously published from the country with similar objectives. Assessing the baseline level of awareness of physicians with regard to malpractice will give us the necessary data to pass recommendations for future preventive measures.

## Methods and Materials

In this study, we conducted a cross-sectional survey of surgeons and senior surgical trainees in different specialties and subspecialties working at various hospitals in Ethiopia. The study was conducted in June 2020. We made a convenience sampling of surgeons and surgical trainees based on their availability taking the list of surgeons from the surgical society of Ethiopia (SSE). The survey was sent online to 230 surgeons and surgical trainees who were available during the study period. A total of 204 respondents completed and submitted the online survey, yielding an 88 percent response rate. Incomplete questionnaires with missing data were discarded. All data from participants were kept confidential by maintaining the study subjects' anonymity and written informed consent was collected before administering the data collection tool. Written ethical clearance letters were obtained from the departmental research and ethics committee.

Google forms were used to collect data anonymously, using a 56-items structured questionnaire. The data collection tool was pretested on an initial sample of ten surgeons and ten surgical trainees. The findings and observations obtained were used to modify the initial questionnaire and the data collection process accordingly.

Data were analyzed using nonparametric statistical methods with the help of SPSS software package 26. Descriptive statistics formed the mainstay of the statistical analysis. Accordingly, frequency tables and summary charts were made.

## Operational Definitions

**Medical malpractice**: Professional negligence by a healthcare provider in which the treatment provided falls below the accepted standard of practice in the medical community and causes injury or death to the patient, with most cases involving medical error (1).

**Medical negligence**: Care which is below the standard expected of the community of health professionals (3).

**Medical adverse event**: An injury that was caused by medical management (rather than the underlying disease) and that prolonged the hospitalization, produced a disability at the time of discharge, or both (3).

**Medical error**: An act of omission or commission in planning or execution that contributes or could contribute to an unintended result (10).

**Informed consent**: The right to the complete information about the course of treatment as well as the risk taken especially during surgical treatment and the right of a shared decision to choose the treatment (11).

## Results

**Socio-demography**: The age of the respondents ranged from 26 to 62 years (mean was 33 years ± 7 years). One hundred sixty-three (79.9%) respondents were males, resulting in a male to female ratio of 3.9:1, with the majority (52.9%) being married.

Ninety-six (47.1%) respondents were surgeons, and 108 (52.9%) were surgical trainees. The majority 66 (61.7%) of the surgeons, had less than five years of experience, 18(16.8%) had five to ten years of experience, and the remaining 23 (21.5%) had more than ten years of experience. Among the surgical trainees, 40 (30%) were in the third year, 55 (50.9%) were in the fourth year, and 13 (12%) were in the fifth year.

One hundred one respondents (49.5%) were from general surgery. Moreover, 30 (14.7%) entrants belonged to plastic surgery, and 25 (12.3%) were from pediatric surgery. Most of the respondents, i.e., 152 (74.5%) practiced in governmental hospitals, while two (6%) respondents worked in a private hospital, and 50 (18.5%) worked in both. Table 1 provides a summary of the sociodemographic data of the respondents.

**Table 1:** Sociodemographic data of respondents

Variable		Value
Age (years)	Range	26–62
	
	Mean	33±7
Gender	Male	163(79.9%)
	Female	41(20.1%)
Marital status	Single	93 (45.6%)
	Married	108 (52.9%)
	Divorced	3(1.5%)
	Widowed	0(0%)
Academic status	Surgeon	96(47.1%)
	Surgical trainee	108(52.9%)

**Knowledge**: To assess knowledge about malpractice, respondents were provided with a list of 15 different forms of malpractice. The mentioned forms of malpractice were defined and described with examples on the data collection tool. Respondents were asked to rate their level of agreement on a Likert scale of 1–5 with regard to whether each of these forms comes under the heading of malpractice. Table 2 provides a summary of the mean Likert scores of the opinions of the respondents for each form of malpractice.

**Table 2:** Opinions of respondents on different forms of malpractice

Type of malpractice	Mean Likert scores
Failure to obtain consent	4.23 ± 1.19
Unnecessary Surgery	3.92 ± 1.14
Misdiagnosis	3.75 ± 1.22
Failure to diagnose	3.79 ± 1.03
Delayed surgical intervention	3.89 ± 1.00
Surgery on wrong patient	4.34 ± 1.24
Surgery on wrong site	4.33 ± 1.25
Unintentional incision	2.90 ± 1.08
Unintended damage to adjacent organs	2.75 ± 1.02
Foreign object left in patient	3.64 ± 1.15
Preventable cosmetic errors	3.49 ± 1.00
Delayed detection and intervention for complications after surgery	3.70 ± 1.04
Anesthesia errors	4.00 ± 1.00
Failure to refer to a specialist/specialized center	3.78 ± 1.01
Failure to break news in entirety	3.60 ± 0.98

Most respondents (71.1%) considered surgery on the wrong patient to be malpractice, followed by surgery on the wrong site (70.1%), failure to obtain consent (59.3%), anesthesia errors (35.8%), and unnecessary surgery (34.3%). On the contrary, unintended damage to adjacent organs (10.8%) was not considered as malpractice by respondents, followed by unintentional incision (8.8%), preventable cosmetic errors (6.4%), unintentionally leaving a foreign object inside a patient (5.4%), and failure to break the news in entirety (2.5%). Overall, only 70.6% of respondents were aware of at least one form of surgical malpractice.

**Attitude**: Respondents were asked a series of 15 questions to assess their attitude towards certain controversial malpractice scenarios. The results are displayed in Table 3.

**Table 3:** Attitudes of respondents regarding malpractice

Question	Response	
	
	Yes	No
Do you believe errors could occur in one's surgical practice?	204 (100%)	0 (0%)
Do you believe a surgeon should disclose his/her errors to their patients?	184 (90.6%)	19 (9.4%)
Will you disclose your error to the patient if the error could lead to major morbidity?	166 (81.4%)	38 (18.6%)
Will you disclose your error to the patient if the error is trivial?	91 (44.8%)	112 (55.2%)
Will you disclose your error to the patient if there is a threat of retaliatory assault?	66 (32%)	138 (68%)
Will you disclose your error to the patient if you find that doing so would place your reputation at stake?	130 (63.7%)	74 (36.3%)
Will you disclose your error to the patient if the patient is not interested in knowing about it?	98 (48%)	106 (52%)
Will you disclose your error to the patient if you feel that they won't be able to understand what you are saying?	85 (41.7%)	119 (58.3%)
Will you disclose your error to the patient if you don't know them very well?	147 (72.1%)	57 (27.9)
Will you disclose near misses	77 (37.7%)	127 (62.3%)
Will you offer to correct your error, free of charge?	185 (90.7%)	19 (9.3%)
Will you offer monetary remuneration if the error is not corrigible?	78 (38.2%)	126 (61.8%)
Will you continue to practice if you find that your senses are weakening, with the resulting negative impact on your career?	60 (29.4%)	144 (70.6%)
Are you willing to apologize for your mistake, if need be?	202 (99%)	2 (1%)
Would you report a colleague if you witness a deliberate act of malpractice?	148 (72.5%)	56 (27.5%)

**Figure 1:**
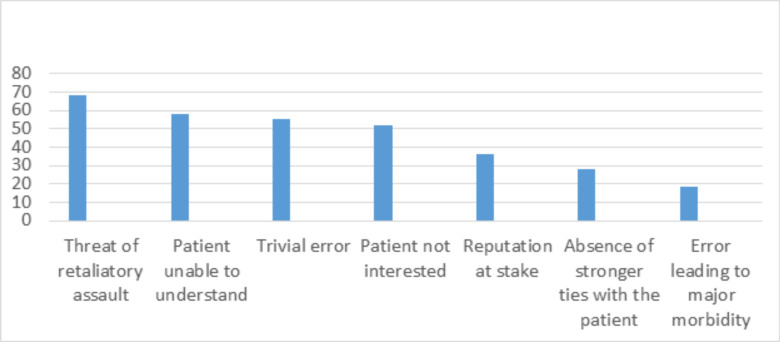
Reasons for not disclosing a medical error

A great proportion of the respondents (90.6%) answered affirmatively when asked, “Do you believe a surgeon should disclose his/her errors to their patients?” In descending order, reported reasons for not disclosing an error were a threat of physical or verbal assault (68%), the patient not being able to understand the nature of the error (58.3%), the error being trivial (55.2%), patient not interested in knowing about the mistake (52%), and reputation being at stake (36.3%). Data is shown in Figure 1. Furthermore, a significant percentage (62.3%) of respondents were not willing to disclose near misses.

Although a significant number of respondents, that is, 185(90.7%), were willing to correct their errors free of charge, only a small group of respondents 78(38.2%) were voluntarily willing to offer monetary remuneration as compensation for their mistake.

Furthermore, 60 (29.4%) respondents answered yes when asked, “Will you continue to practice if you find that your senses are weakening, with the resulting negative impact on your career?” Moreover, 148 (72.5%) respondents reported that they are willing to report a colleague if they witness a deliberate act of malpractice.

**Practices and experiences**: Respondents were asked a series of 15 questions to assess their experience with regard to malpractice. The results are displayed in Table 4.

**Table 4:** Practices and experiences of respondents with regard to malpractice

Question	Response	
	
	Yes	No
Have you ever committed a serious medical error in your surgical practice?	57 (27.9%)	147 (72.1%)
Did you disclose the error to the patient?	34 (59.6%)	23 (40.4%)
Have you ever been physically/verbally assaulted by a patient or their attendants?	120 (59.1%)	84 (41.2%)
Do you have a habit of frequently taking proper informed consent?	177 (86.8%)	27 (13.2%)
Do you keep yourself updated on innovations in medicinal protocols by a regular review of literature?	149 (73%)	55 (27%)
Do you avoid taking a case if you think it is difficult?	129 (63.2%)	75 (36.8%)
Do you have a habit of frequently referring cases to specialists/specialized centers?	151 (74%)	53 (26%)
Do you order more tests than needed, in order to save yourself from liability/accountability?	43 (21.1%)	161 (78.9%)
Do you think that you provide adequate care to your patients?	163 (79.9%)	41 (20.1%)
Have you ever received a legal claim?	10 (4.9%)	194 (95.1%)
If you were sued before, did you win the case?	8 (80%)	2 (20%)
Have you ever been asked to testify in a court of law?	33 (16.2%)	171 (83.2%)

**Figure 2:**
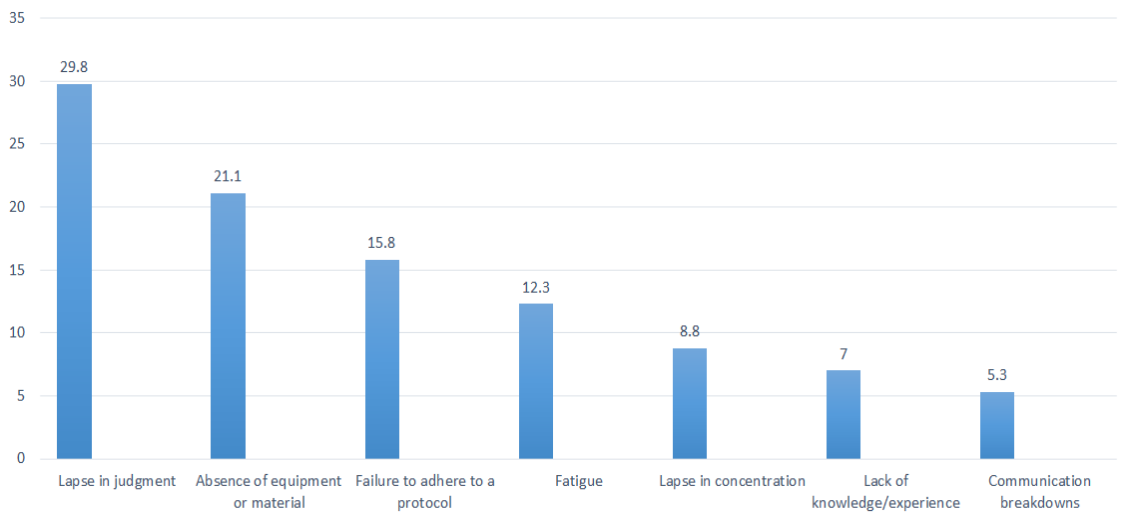
Contributing factors for a medical error

Fifty-seven (27.9%) respondents reported making at least one serious medical error in their careers, but 23 (40.4%) respondents admitted that they did not disclose their mistake to the patient. As demonstrated in figure 2, the top three contributing factors for committing a severe medical error were a lapse in judgment (29.8%), a lack of equipment or material (21.1%), and a failure to adhere to standards and norms (15.8%).

One hundred seventy-seven (86.8%) of the respondents mentioned having a habit of making sure that proper informed consent is collected before surgery. Moreover, one hundred twenty-nine (63.2%) expressed reluctance to accept a case that was deemed difficult. Further, a substantial number of respondents, that is, 120 (59.1%), reported being physically/verbally assaulted by a patient or their attendants at some point in their practice.

Ten (4.9%) respondents reported receiving at least one legal claim during their practice; eight (80%) of them won their case, while those who lost were penalized. Thirty-three (16.2%) of respondents reported testifying in a court of law at least once during their practice.

## Discussion

The number of adverse events is increasing both in developing and developed nations (12–15). Similarly, litigations for malpractice are on the rise (14). However, the overall awareness of health professional to medical malpractice seems to be limited. According to one survey in Ethiopia (Jimma Hospital), most health workers (>60%) believed safety measures in their facility are enough (16). This has the implication of a false sense of adequate safety measures despite the reality might be the opposite.

This study depicts the KAP (Knowledge, Attitude, Practice) of surgeons and surgical trainees toward the concept of medical malpractice. Seventy percent of respondents were aware of at least one form of surgical malpractice. Using the 75% cut-off value commonly applied in KAP studies, this aggregated value falls below the accepted average, implying that our respondents, regardless of academic status or sub-specialty of practice, have a general lack of familiarity with the aforementioned forms of malpractice. A study done by FHPECE revealed a negligence rate of 4.8% (n=125). These includes wrong-site surgeries, misdiagnoses, delayed diagnoses, and unnecessary surgery (17). However, respondents' agreement with these and other forms of malpractice presented was, average. No statistically significant association was found between this poor level of awareness and some important independent variables such as academic status, specialty, years of experience for surgeons, or level of training for surgical trainees. In our study, majority of respondents said they would like to correct their errors for no additional fees. However, only a few were willing to go for financial compensation.

Seventy-five percent of our respondents were not willing to disclose a colleague engaged in an act of malpractice they witnessed during their practice. This observation is contrary to the Ethiopian Food, Medicine and Healthcare Administration and Control Authority (EFMHACA) proclamation 661/209, which states all professionals or other individuals should report the act of malpractice by a colleague (12). EFMHACA proclamation 299/2013 states all health professionals are required to get informed consent from a patient (18). However, only 86.8% of our respondents practiced the habit of frequently taking informed consent. This might indicate that they have not understood the legal implications and provisions associated with their practice.

Fifty-seven (27.9%) respondents reported making at least one medical error in their careers. A similar study from Pakistan reported this ratio to be 36.7% (19). In literatures, a variety of factors have been described attributing to medical errors, such as including fatigue from excessive workload, poor supervision, lack of proper communication, and unavailability of required technology (21–25). In our survey most commonly reported causes for medical errors were lapse in judgment (29.8%), absence of equipment or material (21.1%), and failure to adhere to a protocol (15.8%). Moreover, surgeons' degree of fatigue, a lapse in concentration, lack of knowledge/experience, and communication breakdowns were mentioned. In comparison, Regenbogen et al. reported complex surgeries and technology failures were causes for errors in 61% and 21%, respectively (25). In another study the causes depicted were errors in judgment (72%), teamwork breakdowns (70%), and lack of technical competence (58%) (26).

In our study, failure to adhere to a protocol was mentioned as a cause for medical errors. This point should be emphasized because adherence to safety protocols such as the surgical safety checklist may help in preventing some of these errors. One study reported that using surgical safety checklist might have avoided one-third of errors in surgery (27).

Ten (4.9%) of our respondents were sued at least once during their practice, with three of them being surgical trainees. About 2.4% of malpractice claims (n=125) submitted to EFHPECE within seven years were against surgical trainees (17). The EFMHACA proclamation 661/2009 clearly states that any person in training is responsible for injury inflicted to a patient (28).

Two respondents reported being penalized for medical malpractice they committed, and both paid money to settle damage claims. Apart from legal measures, the EFMHACA reported taking administrative measures like suspension of license, warning letters, and recommendation of additional training (18).

The current surgical training curriculum in the Ethiopian medical setup includes only a one-week (4 ECTs) teaching on “Ethics and Professionalism.” Given the low level of awareness about medical malpractice among surgeons and surgical trainees revealed in this study, consideration should be given to revise the current curriculum with respect to medical ethics, medical malpractice, and litigation issues.

The general outcome of our study showed that surgeons and surgical trainees have poor awareness of the concept of medical malpractice, irrespective of academic status or years of practice. Although some forms of negligence received moderate to high scores, overall awareness of malpractice remained relatively low. The majority were not inclined to be held accountable for their wrongdoings. Complete disclosure of an error was found to be deficient, and a threat of assault was described as the most common reason for failure to disclose an error. The practice of informed consent was also not always followed or understood. A significant proportion of surgeons and surgical trainees were found to be involved in at least one serious medical error during their surgical practice, and several factors were described as contributing to the errors. The results of our study evinced the need for programs aimed at increasing awareness among surgeons and surgical trainees. We recommend more robust ethics and law training modules to surgical trainees, refresher courses to surgeons, and advanced training programs in ethics and law.
